# An evaluation of the implementation of interventions to reduce postoperative infections and optimise antibiotic use across the surgical pathway in India: a mixed-methods exploratory study protocol

**DOI:** 10.1186/s40814-022-01192-z

**Published:** 2022-11-05

**Authors:** Shalini Ahuja, Sanjeev Singh, Esmita Charani, Surya Surendran, Vrinda Nampoothiri, Fabia Edathadathil, Anu George, Andrew Leather, Carolyn Tarrant, Alison Holmes, Nick Sevdalis, Gabriel Birgand

**Affiliations:** 1 Centre for Implementation Science, Health Service and Population Research Department, Institute of Psychiatry, Psychology and Neuroscience, King’s College London, London, UK; 2 Florence Nightingale Faculty of Nursing and Midwifery, King’s College London, London, UK; 3 Department of Infection Control and Epidemiology, Amrita Institute of Medical Sciences, Amrita Vishwa Vidyapeetham University, Kochi, Kerala India; 4 Health Protection Research Unit in Healthcare Associated Infections and Antimicrobial Resistance, Department of Medicine, Imperial College London, London, UK; 5 Division of Infectious Diseases & HIV Medicine, Department of Medicine, Groote Schuur Hospital, University of Cape Town, Cape Town, South Africa; 6 Health Systems and Equity, The George Institute for Global Health, New Delhi, India; 7 Amrita Institute of Medical Sciences, Kochi, India; 8 King’s Centre for Global Health and Health Partnerships, School of Life Course and Population Sciences, King’s College London, London, UK; 9 Department of Health Sciences, University of Leicester, Leicester, UK

**Keywords:** Postoperative infections, Surgical site infections, Antimicrobial resistance, LMICs, India, Implementation

## Abstract

**Introduction:**

Postoperative infections represent a significant burden of disease, demanding antibiotic prescriptions, and are contributing to antimicrobial resistance. The burden of infection as a surgical complication is greater in low- and middle-income countries (LMICs). We report the protocol of a pilot study for the co-design, implementation and evaluation of two infection prevention and control (IPC) and antimicrobial stewardship (AMS) interventions across the surgical pathway in a teaching hospital in India.

**Methods and analysis:**

The two interventions developed following in-depth qualitative enquiry are (i) surveillance and feedback of postoperative infections to optimise the use of antibiotics in two surgical departments (gastrointestinal and cardiovascular and thoracic surgery) and (ii) raising awareness amongst patients, carers and members of public about IPC and AMS. We will conduct a prospective study, formatively evaluating the implementation process of delivering the two co-designed interventions using implementation science frameworks. The study will systematically assess the context of intervention delivery, so that implementation support for the interventions may be adapted to the needs of stakeholders throughout the study. Analysis of implementation logs and interviews with stakeholders upon completion of the implementation period, will offer insights into the perceived acceptability, appropriateness, feasibility and sustainability of the interventions and their implementation support. Implementation costs will be captured descriptively. Feasibility of clinical data collection to investigate effectiveness of interventions will also be assessed for a future larger study. Thematic framework analysis and descriptive statistics will be used to report the qualitative and quantitative data, respectively.

**Strengths and limitations of this study:**

• The paired interventions have been co-designed from their inception with involvement of stakeholders at different stages in the surgical pathway.

• Simultaneous evaluation of implementation and clinical outcomes will inform the development of a future larger study to enable/assess the scalability of interventions

• The study offers a novel combination of implementation theory-informed, stakeholder-driven and clinically relevant evaluation, carried out in the context of a middle-income country hospital.

• The project may not be applicable to every low-resource setting and surgical context due to differences in healthcare systems and cultures. However, the application of implementation science concepts may facilitate transferability and adaptation to other settings.

**Supplementary Information:**

The online version contains supplementary material available at 10.1186/s40814-022-01192-z.

## Key messages

A. What uncertainties existed regarding the feasibility?

- Can the understanding of implementation process assist us in assessing the local context and informing the development of strategies to maximise the adoption and sustainability of IPC and AMS-related interventions?
- Is it possible to gather quantitative clinical data at AMRITA hospital to understand the clinical efficacy of interventions that improve antibiotic use and reduce SSIs in surgical pathways?
- Can surveillance and feedback intervention be delivered as intended at AMRITA hospital?
- Is it possible to produce appropriate and acceptable AMR awareness animation videos for patients’ carers and the members of the public at AMRITA hospital?

B. What are the implications of the feasibility findings for the design of the main study?

- The study informs the type of subsequent trial we run and on the implementation front the nature of strategies we use to implement (and evaluate) each one of the two interventions.

## Introduction

Postoperative infections represent a significant burden of disease [1]. This problem is particularly acute in low- and middle-income countries (LMICs), accounting for up to a third of all healthcare-associated infections (HAI) [2]. When focusing on HAIs occurring in surgical units, 70% of them involve a surgical site infection (SSI), urinary tract infections, or postoperative pneumonia [3]. Up to 60% of surgical patients receive antibiotics postoperatively whilst in hospital, and up to 50% are discharged with a course of antibiotics [4]. Contracting an SSI increases antibiotic use seven-fold, compared to uneventful postoperative recovery [5]. The risk of postoperative infection can be managed and mitigated through a series of pre- and post-operative preventive and curative measures [6, 7]. The compliance with those measures is critical to reduce reliance on antibiotics.

The capacity of healthcare systems to deliver safe surgery depends on the availability of effective antibiotics, particularly for use prophylactically to reduce the risk of infection. In surgical patients, the overuse and inappropriate use of antibiotics both for prophylaxis and to treat postoperative infections remain areas of additional concern [8]. The associated growth and spread of antimicrobial resistance (AMR) undermine not only current surgical treatments but also potential advances and innovation in surgical interventions [9]. AMR poses a societal-level threat, and there is evidence that this threat has already impacted surgical care in LMICs [10].

Contextual factors shape infection prevention and control (IPC) practices, and antibiotic prescribing decision-making in hospital settings, including in surgical care [11]. Interventions to improve surgical safety, such as the WHO surgical safety checklist, have been implemented internationally [12]. The success of such interventions is, however highly context-dependent [13], and influenced by economic, cultural and social factors including role, identity and hierarchies within healthcare teams [14]. Such interventions consider and address a single point on the patient’s pathway. While we know that hierarchies and peer behaviours influence prescribing behaviours [15] and IPC practices, less is known about involvement of antimicrobial stewardship (AMS) teams within multidisciplinary surgical teams, and about communication with professionals across organisational boundaries along the surgical pathways [16]. The role of the patient is also un-explored, and potential strategies for empowering patients in IPC and antibiotic use [17] have not been extended to the surgical context [18].

In LMICs, numerous factors further complicate the access to, and the safety of, surgery. These include access to surgical treatment and care and the availability of surgical and anaesthetic providers, financial and geographical restrictions, cultural beliefs, poor education and lack of structural and human resources, particularly microbiologists and nurses [19]. These limitations can have consequences for the effectiveness of IPC in perioperative care and the sustainability of interventions. From a curative point of view, access to antibiotics (including the actual availability of appropriate antibiotics, and out-of-pocket costs) may be limited in some LMICs, or antibiotics may be widely available (e.g. within the community, without the need for appropriate prescription, to those who can afford them) [20]. Such contextual factors may lead either to lack of antibiotics where they are needed, or inappropriate overuse and/or unsuitable choice of drug, dose and treatment duration [21].

Evidence on the use of conceptual frameworks and theories explaining why an intervention worked and whether it worked as intended comes from high-income countries. Research on informed, effective social and behavioural interventions is particularly important in resource-limited settings to bring about efficiencies at the organisational level, but also to address the complexity of providing evidence-based care [22, 23]. It is therefore necessary to explore and understand why and which evidence-based interventions work and for whom [24]. The field of implementation science can help us understand these local contexts, and further inform the development of strategies to maximise the uptake and sustainability of IPC and AMS-related interventions [25].

### Study aims and objectives

We report the protocol of an interdisciplinary pilot study aiming to evaluate the implementation of two IPC and AMS interventions across the gastrointestinal (GI) and cardiovascular and thoracic surgery (CVTS) care pathways at Amrita Institute of Medical Sciences (AIMS) in Kerala, India.

The primary aim of the study is to map the steps needed to optimise the implementation process for both interventions, via formative and iterative evaluation of their acceptability, appropriateness, feasibility and sustainability. Additionally, we will estimate the implementation costs to enable the potential for scale-up in other hospitals and other LMIC settings.

The secondary aim of this study is to establish the feasibility of collecting quantitative clinical data regarding the effectiveness of two interventions in improving use of antibiotics, reducing SSIs in the study pathways and improving patients’ and carers’ awareness of IPC measures and AMR—for inclusion in a subsequent larger-scale study.

## Methods and analysis

### Study design

The study will apply a prospective pilot evaluation approach, formative assessment of the implementation process (to address feasibility, appropriateness, acceptability and sustainability of the interventions and their implementation support strategies) and the feasibility of collecting further quantitative data to assess clinical impact.

For the formative evaluation of implementation, we plan to apply a qualitative methodology incorporating a variety of data collection techniques. These include semi-structured interviews with stakeholders and analysis of monthly implementation logs collected by the research team and observations collected by the implementation team. The implementation logs and team observations will be gathered using embedded research principles, in which researchers and implementors collaborate to generate learning from the implementation process that can be used to improve it, and rapid ethnography and auto-ethnography, in which team members keep field notes and observations reflecting their perceptions and experiences of the implementation process [26]. These materials will be analysed in conjunction with the data from interviews with the study stakeholders (including frontline staff and patients).

For the remaining feasibility assessments, we will use a set of standard clinical outcomes, collected through the hospital’s existing administrative and patient records. Different sources were used to collect data on clinical outcomes. While manual checks and clinical assessment by infection prevention nurses enabled surveillance of surgical site infections and bloodstream infections, audits and pharmacy records were used to collect data from Antimicrobial stewardship team and WHO AWaRe index for antibiotic prescription.

### Criteria for determining success of feasibility

- The qualitative study is of value in identifying actual, rather than anticipated, facilitators and barriers to the implementation and sustainment of the two interventions which will help in the next stage of pilot trial design. This is essentially a very first step in determining how best we can intervene in this setting, so a formative study.
- Data collection mechanisms are being established to collect clinical data for this formative study. As above, the stage of the study is quite an early one, so we are supporting data collection for the purposes of the study and aiming to find out what additional support structured may be required for future study/ies. As such, we did not set out quantitative criteria for determining feasibility here—we are not at that stage of study development yet. Although the study’s data collection is still ongoing, preliminary evidence points to the feasibility of collecting the data using manual checks, audit and hospital information systems.

### Participants, interventions and outcomes

#### Study setting

AIMS, a tertiary care hospital in India, has robust IPC and AMS teams [20, 27]. However, patient and carer participation has not yet been incorporated in the design or implementation of programme measures [28].

AIMS is working to optimise antibiotic usage in surgical care through the ASPIRES (Antibiotic use across Surgical Pathways—Investigating, Redesigning and Evaluating Systems) programme. The ASPIRES programme includes preventative measures to reduce the risk of infection and optimise prophylactic and therapeutic antibiotic use and to develop evidence- and user-informed implementation strategies tailored for effective implementation and sustainability (for more details: https://www.imperial.ac.uk/arc/aspires/).

#### Participant’s eligibility criteria

The eligible clinical population will comprise adults over the age of 18 who are treated as elective or emergency general surgery patients and are assessed as high risk for SSIs, identified as having developed an SSI and/or have received antibiotics for an active infection while under the care of the GI or CVTS surgical teams. Patients undergoing transplants, patients under the age of 18 and patients not under the care of the GI and CVTS surgical teams will be excluded from participation. For staff participants, we will employ purposive sampling to identify key informants for the semi-structured interviews. Participation will be voluntary.

#### Interventions

In the first phase of the ASPIRES programme, we conducted a three-step process to identify key interventions. A number of qualitative interviews (*n* = 25) and expert consultations (*n* = 5) yielded key areas of intervention in relation to AMS and IPC. A list of potential interventions drawn up from the qualitative outputs was presented during prioritisation workshops held with hospital staff to prioritise two interventions for implementation. The top two interventions are detailed below:

##### First intervention—enhanced patient and carer involvement in AMS and IPC

Educating patients and their carers about the appropriate use of antibiotics is an important measure in mitigating AMR, particularly in relation to health-seeking behaviours. In the AIMS setting, where out-of-pocket expenses are associated with care, pressure and demand for services from family and carers, a need was identified to explore the role of the patient and carer in IPC. Furthermore, in India, where there is no precedent for patient and public involvement, this intervention in itself is an innovation.

Qualitative research underpinning this work from the ASPIRES research team suggests that hierarchical decision-making in surgical care and an unrecognised carer’s role prevent effective patient participation in any given cultural context [28, 29]. Literature also highlights that postoperative recovery including wound care entails patient and carer participation and cooperation and is therefore critical to positive surgical outcomes [18].

Moreover, the contextual analysis found potential opportunities for furthering the role of the medical social workers in education and training on AMS and IPC [30]. Bespoke animated videos on IPC practices, AMS and AMR will be developed and played in the general wards to raise awareness amongst patients, carers and other members of the public. As booklets/leaflets on IPC practices are already in use in the cardiovascular surgery department, key champions, such as admission staff, will be involved in distributing leaflets detailing best IPC practices to GI patients. Involvement, participation and engagement of patients and carers will be critical components of developing educational materials.

**Fig. 1:**
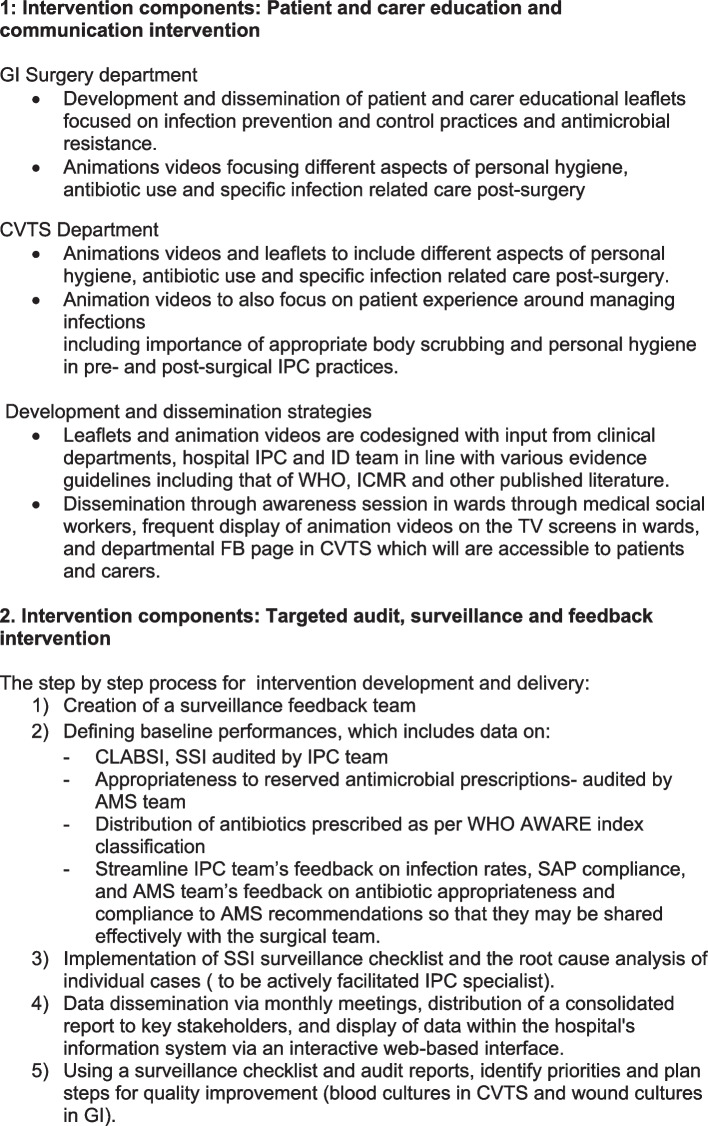
Intervention components

This intervention component aims to implement the educational multimodal intervention for patients and carers to optimise postoperative recovery and care and to measure the effect of the intervention on knowledge, practices and attitudes of patients and carers in relation to wound care and antibiotic use in the surgical pathway (Fig. 1).

##### Second intervention—targeted audit, surveillance and feedback to surgical teams

Earlier qualitative research within the ASPIRES programme established that several stakeholders, including surgeons, were not aware about existing surveillance and reported that they were not receiving any feedback from AMS or IPC teams [31]. Co-design workshops, in which this initial evidence was presented alongside other evidence on IPC and AMS interventions within the surgical care pathway were carried out with AIMS participants [31]. A scoping review was performed to assess the body of literature in this field and further inform the co-design workshops [32].

The first component of the intervention involves ensuring surgeons receive real-time feedback about IPC and AMS audits. This will take into account goal setting, action plans, benchmarking and visual aids [33]. The second component of the intervention relies on implementing a comprehensive plan to feedback surveillance (and audit) data collected from operating rooms, intensive care units, surgical wards and the antimicrobial stewardship team to key stakeholders by keeping emphasis on content and process of delivery of feedback, goal setting and action planning (Fig. 1).

#### Development of implementation strategies for the two interventions

Several implementation frameworks support the conduct of the study and the selection of implementation strategies that support the delivery of interventions. To date, the ERIC implementation strategies framework is the best established framework for the identification, analysis and selection of strategies that drive the implementation and sustainability of evidence-based clinical interventions, such as those planned within ASPIRES [34]. Seventy-three discrete implementation strategies were proposed, then arranged into nine thematic categories. The ERIC framework mapped these strategies against barriers to implementation through qualitative work with stakeholders and prospective evaluations. The strategies were identified through inputs from other studies conducted by ASPIRES qualitative research and four stakeholder workshops and will be refined through prospective evaluations of intervention implementation in situ [29]. In collaboration with the local team in India (clinicians and researchers), implementation support ‘bundles’ will be designed to support the application of the interventions.

The local research team in the hospitals have been trained to audit cultures, streamline feedback, optimise communication and ensure patient and carer participation and involvement. Meanwhile, the core implementation team consists of surgeons, clinical and surgical nurses and operating theatre staff, IPC and AMS team members, MSWs, patients and carers.

#### Theoretical frameworks for evaluation

Exploration, Preparation, Implementation, Sustainment (EPIS) framework [35] was selected to support the conceptualisation and subsequent analysis and reporting of the process of implementing the two interventions at AIMS. The four phases that the framework suggests—namely Exploration, Preparation, Implementation, and Sustainment—will be used to map the process of implementation and the progress made within the study. The framework will assist us in describing the implementation process, understanding the external and internal context, as well as other intentional and unintended consequences that bridge the gap between an intervention and its use in practice.

In addition to the EPIS framework, the taxonomy of outcomes of an implementation effort proposed by Proctor and colleagues [36] offers a structured framework for us to consider the introduction of the two interventions at Amrita and the progress, over the timeline of the study, of the implementation. The taxonomy includes the acceptability, feasibility and appropriateness of an implementation strategy; the stakeholders’ intention to adopt it; the fidelity of its application; its uptake; its sustainability; and its costs. We will address several of these outcomes while implementing the intervention ‘bundles’ qualitatively, through interviews with stakeholders and ethnographic methods (implementation logs and field notes) to both determine and support the success of the implementation throughout the timeline of the study—as the implementation analysis is of a formative nature (Additional file 1: Appendix 1).

#### Clinical outcomes

As part of the pilot study, we will be collecting clinical outcomes to evaluate whether they are feasible to collect for the purposes of a future larger study (Tables 1 and 2). For this purpose, we will collect surgical site infection (SSI), blood stream infections and mortality, appropriateness of prescribing reserve antibiotics; compliance to AMS recommendation culture appropriateness, adherence to SSI prevention guidelines and improvement in the distribution of antibiotics prescribed as per WHO AWaRe (Access, Watch and Reserve) index classification.

**Table 1:** Summary of the outcome measures for the two interventions (patient and carer education and communication intervention and audit, surveillance and feedback intervention)

Outcome (Patient/ Service Implementation)	Measurement method(s) (e.g. observations, surveys, routinely collected data)	Level of measurement (i.e. patient, provider, surgical dept or hospital level)	Measurement time point(s)
**Patient and carer education and communication intervention**			
Patient and carer involvement and engagement in the content and the inception of the intervention	Qualitative interviews of patients and carers who were involved in the intervention. Structured questionnaires to assess the knowledge attitude and behaviour of patients and cares towards the use of the intervention.	CVTS, GI	Feb-Mar 2021
**Audit, surveillance and feedback intervention**			
1. Clinical: SSI, BSI and mortality; Appropriateness of prescribing reserve antibiotics; Compliance to AMS recommendation	Routinely collected	CVTS, GI	Dec2020 Jan 2021
2. Culture appropriateness, Adherence to SSI prevention guidelines.	Audit Point prevalence surveys	CVTS, GI Wound culture data from GI dept Blood culture data from CVTS dept	May 2021–Jan 2022 Collected bi-monthly
3. Improvement in the distribution of antibiotics prescribed as per WHO AWaRe (Access, Watch and Reserve) index classification.	Audit	CVTS, GI	May 2021 Endline (Jan 2021)
4. Process outcomes: Mechanism employed for the interpretation and feedback of clinical indicators, and how goals are set and actions planned for the SSI prevention and the antimicrobial stewardship, and the level of achievement of goals	Monthly: -Analysis of transcripts from monthly meetings with the surgical team -Analysis of meeting notes with recurrent questions	CVTS, GI	Monthly implementation log May 2021–Jan 2022

*CVTS* cardiovascular and thoracic surgery, *GI* gastrointestinal surgery

**Table 2:** Summary of implementation outcomes

Pilot testing of implementation outcomes to assess the delivery of mechanisms for the two interventions				
Methods	Design	Study population and sample size		
		Local research and implementation teams	Surgeons, OR staff, nursing staff, AMS and IPC staff	Patients, carers and members of the public
In-depth interviews	Qualitative (at the end of the implementation period)	Acceptability, appropriateness, feasibility, sustainability, unintended consequences, contextual factors and implementation strategies (*n* = 20)		Acceptability, appropriateness, feasibility, sustainability unintended consequences, contextual factors and implementation strategies (n=5)
Rapid ethnographic assessments (implementation log + observations)	Observations Meeting notes	Implementation process		NA
Descriptive analysis	Cost template	Implementation cost		NA

Process outcomes: Mechanism employed for the interpretation and feedback of clinical indicators, how goals are set and actions planned for the SSI prevention and the antimicrobial stewardship, and the level of achievement of goals.

### Study timeline

**Fig. 2:**
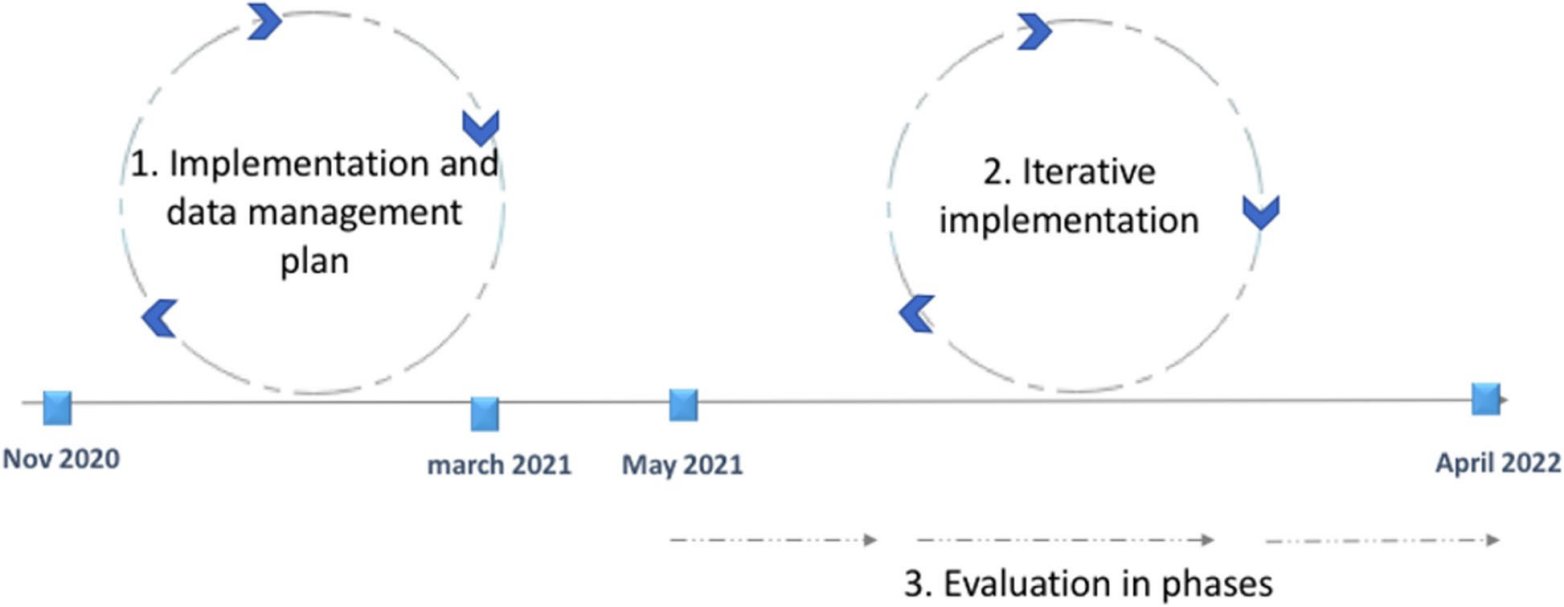
Study timeline

The study will last 15 months and will be divided into three parts. First, the implementation and data management plans will be developed. Second, the interventions will be implemented in GI and CVTS. Finally, the data about implementation process and outcomes and clinical data will be collected and analysed (Fig. 2).

### Sample size

#### Intervention 1: patient and carer involvement

We anticipate we will conduct approximately 10–12 interviews with carers and staff to inform the development of educational materials. In addition, 20 structured questionnaires (or when data saturation is achieved) will be conducted to measure the effect of the intervention on knowledge, practices and attitudes of patients and carers in relation to wound care and antibiotic use in the surgical pathway. Existing guidance suggests that saturation in the themes that appear across participant interviews starts to appear after 6 interviews, and suggests that researcher produces approx. twice that number as a guidance [37].

#### Intervention 2: targeted audit, surveillance and feedback

GI and CVTS surgery will serve as a tracer, firstly because of the high risk of SSI (GI) and secondly because of the high individual burden associated with SSI. This will help in gaining insights and developing solutions that optimise antimicrobial stewardship and infection control practices in all the surgical specialities.

Surveillance of SSIs, bloodstream infections, appropriateness of reserved antimicrobial prescribing and compliance to AMS recommendations will rely on the current routine practices at Amrita. Local research teams will collect mortality rate data. We will collect and analyse all data from the monitoring forms during the period of the study. At the time of data entry, we will anonymise records (using a system of coding all unique entries).

We plan to include all GI surgical procedure representing 1000 to 1500 procedures per year and 40 to 60 SSI. In CVTS, we will focus on median sternotomy (CABG, valve repair) representing around 500 procedures per year. Appropriateness of SAP for all these procedures. There are around 280 antibiotic prescriptions per year at Amrita in the two departments. There are 30 to 40 BSI per year in two departments.

For the point prevalence survey, we expect 15–30 data collection forms on culture appropriateness will be collected per speciality. Finally, we will make transcripts and take notes during each monthly feedback meeting.

The sample size for the definitive trial will be determined later when a feasibility pilot is conducted.

#### Implementation of interventions

The sample comprises five local research teams and healthcare providers from the core implementation team (surgeons, nurses, operating room staff, Medical Social Workers (MSWs), pharmacists from AMS and users from IPC team). Following the establishment of the two interventions, approximately 15–30 interviews will be conducted with the local research team (i.e. the embedded researchers), healthcare workers, patients and carers to gain a thorough understanding of the implementation process. Interviews will be conducted until saturation is achieved which is typically estimated to be reached within 10–12 interviews for homogeneous topics [37].

### Recruitment

For some outcome assessments, the research team will directly sample or interview patients and carers. In other instances, we will review the patient data collected by AMS and IPC teams. These might include a listing of patients based on infection rates and antibiotic records of patients undergoing surgery within the GI and CVTS departments.

### Study and data collection procedures

Data collection will begin at the same time as the implementation—the implementation log and fieldnotes will be recorded throughout. Fieldwork will include interviews at the hospital and the review of completed records (such as infection rates and antibiotic appropriateness data). Data collection will rely on (i) audiotaped interviews, (ii) completed implementation logs and fieldnotes by the study personnel and (iii) completed observation forms (for recording IPC and AMS data).

#### Description of analysis

We will audiotape and transcribe the qualitative interviews. Interviews conducted in Malayalam will be translated into English, and back translation checks will be applied. NVivo-11 software will facilitate thematic extraction and thematic framework analysis for all qualitative interview data. Following familiarisation with the texts and in vivo coding, we will adapt the framework to include additional themes derived inductively from the dataset. Implementation logs and fieldnotes by the embedded researchers will be mapped onto the EPIS framework to understand the implementation process. It will be difficult to establish associations between interventions and their effects on clinical data such as infection rates, the appropriateness of sending blood and urine cultures, and an improvement in antibiotic distribution according to the WHO AWaRe (Access, Watch, and Reserve) index classification. As a result, routine clinical data will be recorded and analysed using descriptive statistics. SSI rates will be assessed using the INICC surveillance methods and stratified by surgical procedure types [38]. Antibiotic appropriateness will follow the WHO AWARE index guidelines. BSI will be classified by portal of entry and by organisms. Mortality will be assessed for patients that undergone GI and CVTS procedures. Point Prevalence Survey data collection sheet for diagnostic stewardship (blood culture and urine culture) will assess the sampling method and the traceability (request form with indication, etc.) according to local guidelines.

### Ethics and dissemination

The study has been approved by the following: Imperial College Research Ethics Committee, Amrita Research Ethics Committee (ECASM-AIMS-2021-014). Results will be made available to healthcare professionals, patients, their caregivers, the funders, scientific societies and other researchers through scientific publications in peer-reviewed journals.

## Discussion

Our study will methodically understand the process of delivering social and behavioural interventions in hospital settings, including interventions aimed at patients and carers which are relatively rare in the low- and middle-income context. The feasibility and possible sustainability of evidence-based, context-driven health interventions aimed at improving surgical care will also be investigated, as well as the likelihood of collecting quantitative clinical data for future trials.

In LMICs, infections are the most common postoperative complications, with patients more likely to be infected with antibiotic-resistant bacteria and [10] twice as likely to die due to infection-related complications than patients in high-income countries [1]. In such settings, additional problems often occur, for example, limited access to surgical treatment and care, financial restrictions or conflicting cultural beliefs [19]. To date, IPC and AMS programmes in hospitals have mainly focused on medical specialties in HICs and taken a one-size-fits-all approach. The contextual factors of the surgical pathway and LMICs settings are critical to consider when designing IPC and AMS interventions in perioperative care. The design and methodology of this study protocol is adapted to the cumulated challenges described. Effective and sustainable integration of optimised IPC and AMS in surgical pathways in LMICs needs an approach that recognises the structural foundations to support staff to be able to change their behaviours and organisational support to enable maintenance of such changes.

The co-design approach that we will take balances power between researchers, providers and service users in decision-making, increasing perceived ownership, and thus enhancing fidelity, uptake and sustainability of interventions. The gastrointestinal and cardiovascular and thoracic surgical pathways will serve as a tracer to gain insight and develop solutions which optimise AMS and IPC practices in all surgical pathways in similar settings. The range of surgical specialities and contexts will provide variation along the axes of cultures and attitudes, infection and AMR burden, and opportunities for innovation. Cross-specialty qualitative comparisons will allow us to achieve rich learning and open up bi-directional impact and innovation pathways.

The intended beneficiaries from this proposed study are public/patients/carers; medical, surgical and health professional staff; associations; and networks. The short-term benefits of the research will likely impact healthcare providers and managers, who will benefit from enhanced ways of working and using efficient behavioural interventions to optimise IPC practices and antibiotic use. Policy-makers will benefit by having access to research- and user-informed approaches to tackle HAI and AMR, especially in the longer term. After the two interventions’ implementation plans are crystallised, an IT platform will be created to cascade the delivery and outcomes of the two interventions, with the goal of ensuring project legacy and sustainability. Ultimately, the outputs of this work will inform large-scale implementation trials, to prospectively evaluate clinical effectiveness and implementation success of a small number of piloted interventions that can be implemented and have the capability to reduce HAI and AMR. Understanding the nature of these interventions’ effectiveness data, the extent to which their delivery plans need to be tweaked, understanding of implementation barriers and facilitators, and evidence or clarity of previous implementation strategies may all aid in deciding the type of hybrid designs for future trials [39].

Several limitations are anticipated. First, this study protocol offers small-scale pilot evaluation that will require follow-up by a larger study, e.g. a hybrid effectiveness-implementation or pure implementation trial. Second, the project may not be applicable to all low-resource settings and surgical contexts due to differences in healthcare systems and contexts. Third, the study does not explore the combined effects of the two interventions and how the two interventions work together. The evidence regarding the effectiveness of clubbing implementation strategies and different innovations is only emerging and it is overall variable—including in the way strategies are used and reported. Nevertheless, the application of implementation science concepts in understanding the process of implementing the two interventions may facilitate transferability and adaptation to other settings.

## Conclusion

This pilot study protocol aims to employ innovative methods of implementation science to address the critical contextual factors to improve postoperative infection prevention and antibiotic use across the surgical pathway in India, a LMIC country. The underlying premise is that the research is driven by the stakeholders’ needs and situated within existing quality improvement approaches within the local surgical pathways and wider hospital. The findings will inform larger-scale studies and can also be used to support implementation of hospital-wide ongoing IPC and AMS interventions and programmes and foster engagement with locally embedded researchers.

## Supplementary Information

**Additional file 1: Appendix 1.** Implementation science: basic concepts and definitions. **Appendix 2.** Summary of characteristics of studies meeting final (stage 2) inclusion criteria. **Appendix 3.** The search strategy for the Medline/EMBASE database. **Appendix 4.** Eight gold-standard implementation outcomes as defined by the implementation science evidence-base [19] and adopted by the WHO [16]. **Appendix 5.** Definitions of implementation strategies, ERIC framework [17].

## Data Availability

Data and materials can be requested from the corresponding author.
